# Cutaneous Cryptococcosis Manifested as a Large Cystic Mass: A Rare Manifestation of Cryptococcus Infection

**DOI:** 10.7759/cureus.58040

**Published:** 2024-04-11

**Authors:** Zain Amar, Muneeb Rehman, Yasir Ahmed

**Affiliations:** 1 Infectious Diseases, Ascension St. John Hospital, Tulsa, USA; 2 Infectious Diseases, Ascension St. John Medical Center, Tulsa, USA

**Keywords:** cryptococcal meningitis, cryptococcal skin lesion, cavitary lung lesion, cystic mass, cryptococcus neoformans infection, disseminated cryptococcus

## Abstract

Cryptococcus infection is an invasive fungal infection common in immunocompromised hosts, especially in organ transplant recipients and in patients with HIV. Its presentation varies from localized skin lesions to systemic disseminated infection involving the lungs and the central nervous system (CNS). We present the case of a 50-year-old woman with diabetes mellitus type 2 (DM-2), end-stage renal disease (ESRD) status post deceased donor kidney transplantation seven and a half years ago who presented with a low-grade fever, cough, nausea, vomiting, and a large cystic mass on the right foot. A CT scan of the chest showed a 14 mm cavitary lesion in the middle lobe of the right lung. Serum and cerebrospinal fluid cryptococcal antigens were detected. MRI of the right foot showed a large multilocular lobulated septated cystic mass. Histopathology showed cryptococcus; the diagnosis was made as disseminated cryptococcus infection. She was treated with antifungal therapy successfully. A large cutaneous cystic mass is a rare cutaneous presentation of cryptococcus infection; clinicians should keep it in the differential diagnosis, especially in transplant recipient patients.

## Introduction

Cryptococcosis fungal infection is caused by an encapsulated yeast,* Cryptococcus neoformans, and Cryptococcus Gattii *species, associated with significant morbidity and mortality [1]. Humans can get the infection by inhaling pigeon excreta, soil, dropping, or even fruits, which may harbor cryptococcus fungus. It can cause localized cutaneous and systemic fungal infection [2]. Cutaneous cryptococcus infection results either from localized invasion through an open wound or as a manifestation of systemic disseminated infection [3]. Cutaneous cryptococcus infection presents with a wide variety of skin lesions, ranging from papules, maculopapular lesions with an ulcerated center or violaceous nodular lesions, cellulitis with pruritic rash, cold abscesses, umbilical type lesions, or rarely large cystic masses. In disseminated infection, the lungs and CNS are involved frequently [4,5]. We present a rare case of a large cystic cryptococcus cutaneous mass with disseminated infection involving the lungs and CNS in a kidney transplant recipient patient.

## Case presentation

**Figure 1:**
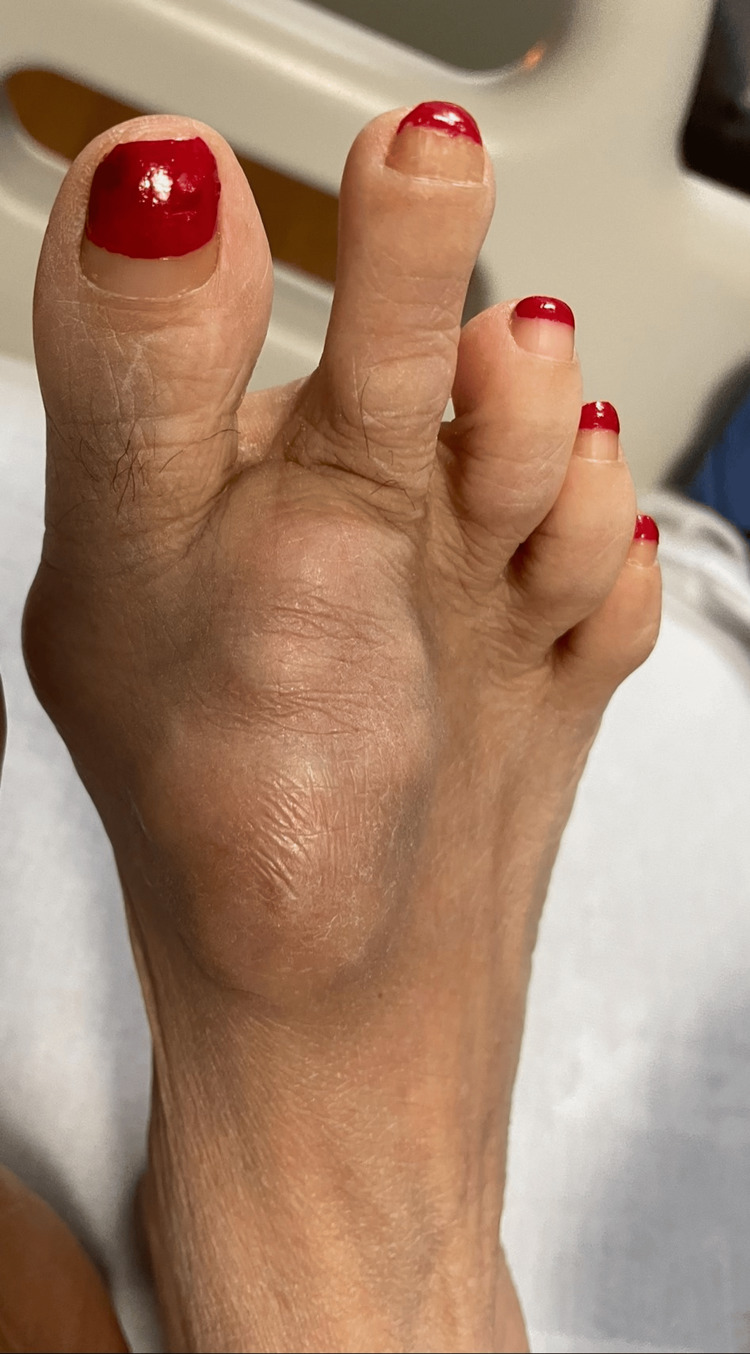
Right foot A large non-tender mass of 6 x 4 cm in size on the dorsum of the right foot.

A 50-year-old Hispanic woman with a medical history of hypertension, diabetes mellitus type 2 (DM-2), ESRD status post deceased donor renal transplantation seven and half years ago, currently on immunosuppressive medications including tacrolimus, mycophenolate, and prednisone. She presented to the emergency room with complaints of nausea, vomiting, cough with clear sputum production, mild headache, fatigue, and subjective fever for one week. She also reported a painless mass on her right foot which had been growing slowly over the past six months. She had no occupational or bird excreta exposure. She did not recall foot trauma, an open foot wound, or an ulcer. Physical examination revealed a temperature of 38°C, a heart rate of 96 beats/minute, a respiratory rate of 18/ minute, and a blood pressure of 128/77mmHg. A large non-tender mass of 6 x 4 cm in size on the dorsum of the right foot was palpable (Figure 1).

**Figure 2:**
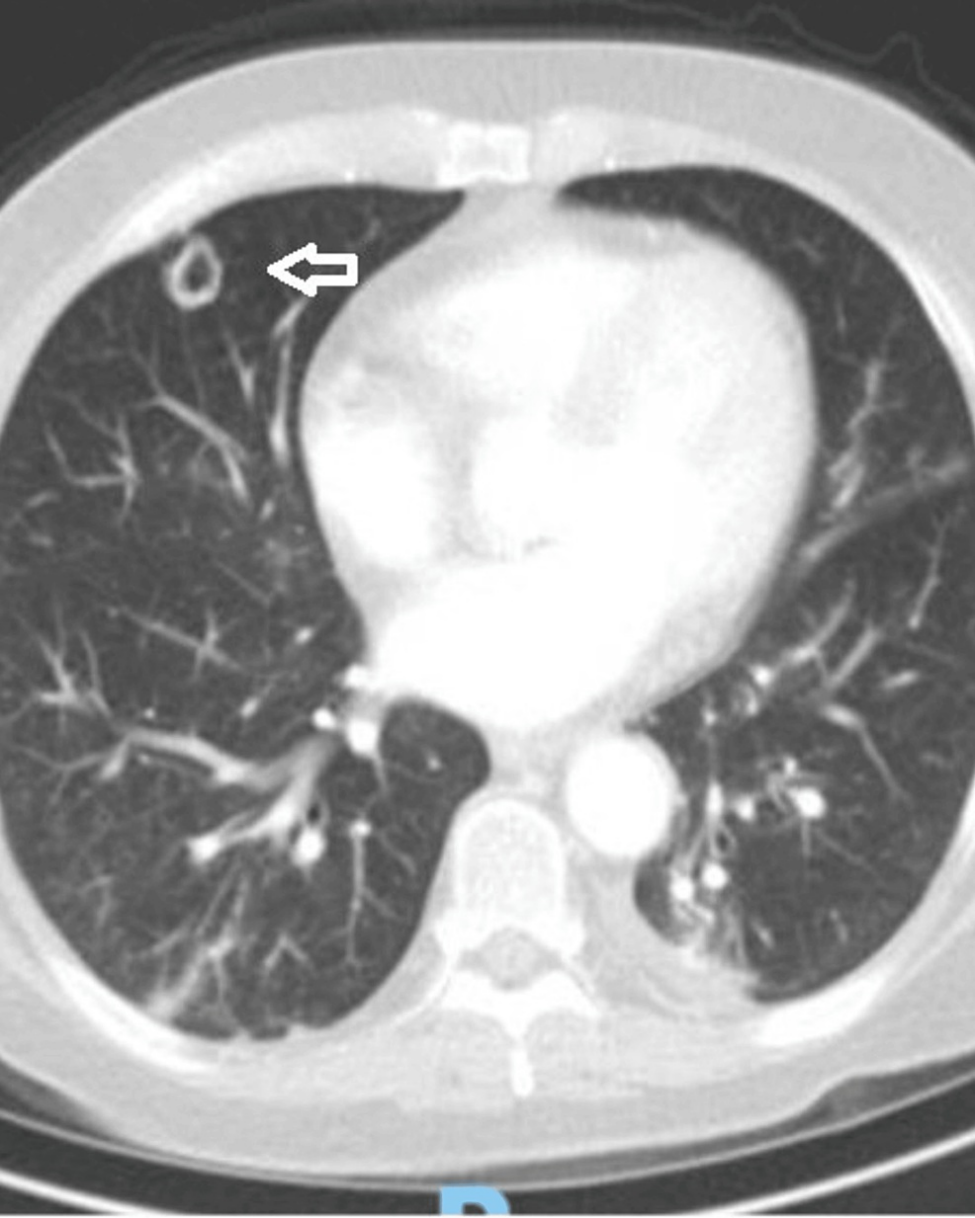
CT chest scan without contrast A computed tomography (CT) scan of the chest showed a 14-mm cavitary lesion in the middle lobe of the right lung (arrow).

The routine blood tests were normal except for the serum sodium which was low (Table 1). The chest X-ray showed a nodule in the right middle lobe. A computed tomography (CT) chest without contrast showed a 14-mm cavitary lesion in the right middle lobe and alveolar opacities in both lower lobes of the lungs suggestive of pneumonia (Figure 2).

**Table 1:** Blood and CSF laboratory data BUN: Blood urea nitrogen, CSF: Cerebrospinal fluid, TB: Tuberculosis, CrAg: Cryptococcal antigen, ND: Not detected.

Table 1 Blood and CSF Laboratory Data		
Study	Value	Normal ranges
White blood cell count, X10^3^/μL	11	4-11
Hemoglobin , g/dL	10.6	11.6-15.7
Platelets, X10^3^/μL	178	150-450
BUN, mg/dL	12	5-20
Creatinine, mg/dL	1	0.5-1.10
Sodium, mmol/L	131	135-145
Hemoglobin A1C %	7.5	4-5.6
Serum CrAg	1:640	ND
Seum TB QuantiFERON	Negative	Negative
CSF CrAg	1:640	ND
CSF India ink stain	Negative	Negative
CSF Opening pressure cmH2O	22.5	6-25
CSF White blood cells	14	0-7
CSF Lymphocytes,%	100	0-70
CSF Protein, mg/dL	167	15-45
CSF Glucose, mg/dL	64	50-80
Serum Glucose, mg/dL	131	70-100
Serum CrAg at 15 months treatment	1:10	ND

**Figure 3:**
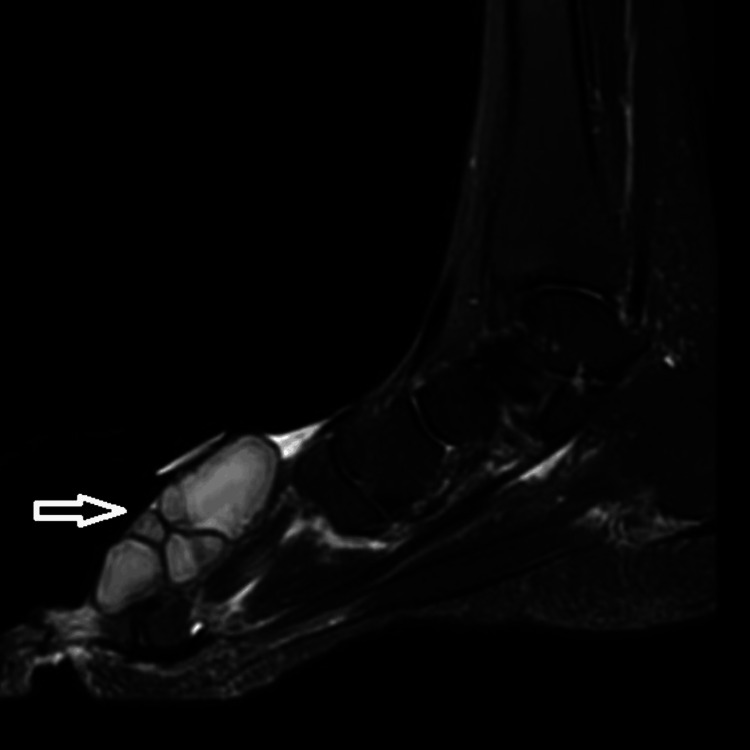
MRI of the right foot A large multilocular lobulated septated cystic mass size of 6 x 6 cm at the first-second intermetatarsal interval between metatarsal heads (arrow) suspicious for large ganglion cyst vs synovial sarcoma.

Magnetic resonant image (MRI) of the right foot revealed a large multilocular lobulated septate cystic mass of 6 x 6 cm in size at the first-second intermetatarsal interval between metatarsal heads suspicious of large ganglion cyst vs synovial sarcoma (Figure 3).

**Figure 4:**
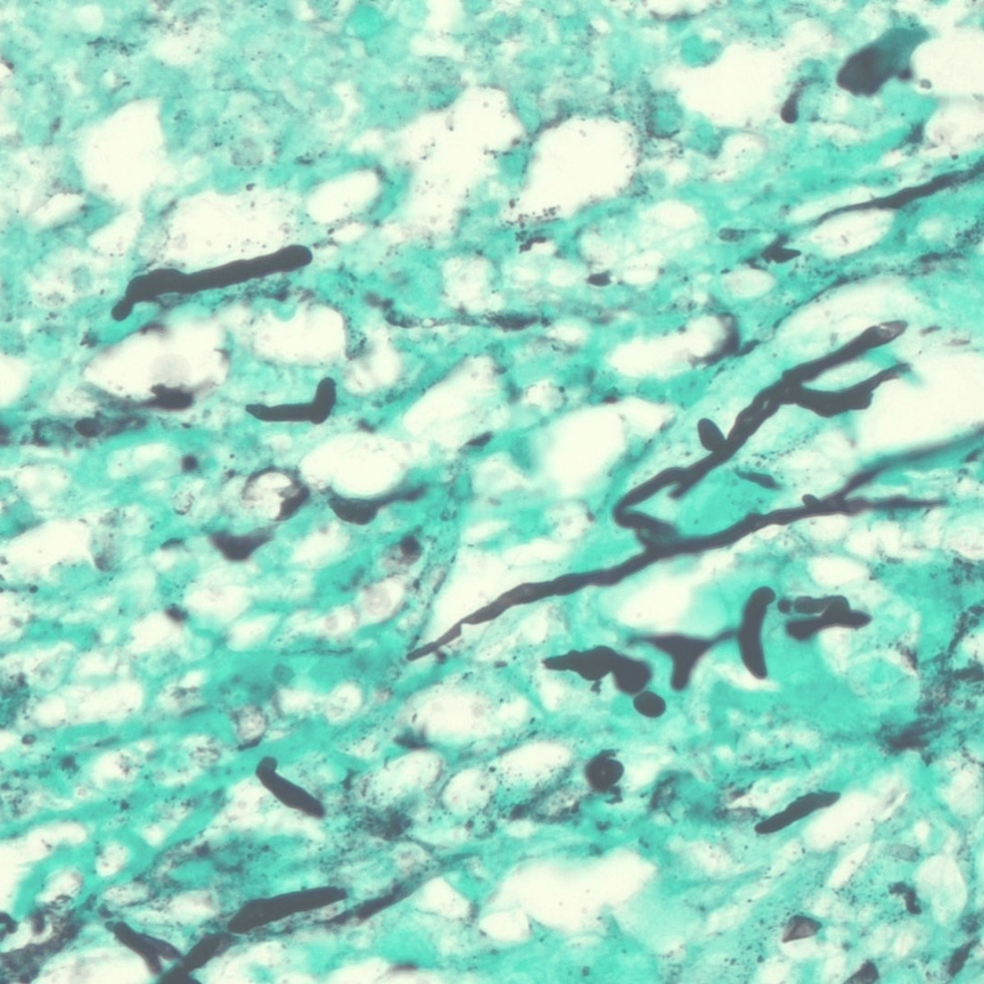
Right foot cystic lesion histopathology Fibroadipose tissue with abscess formation and granulomatous, Grocott–Gömöri's methenamine fungal stain (GMS) positive and shows yeast form fungi with pseudo-hyphae favor Cryptococcus infection.

Blood cultures showed no growth. Serum Cryptococcal antigen detected very high titer; results are shown in Table 1. Other fungal serologies and HIV 4th generation enzyme-linked immunosorbent assay (ELISA) tests were negative. MRI of the brain did not show any lesions or hydrocephalus. The patient underwent a spinal tap. Cerebrospinal spinal fluid (CSF) studies were abnormal as shown in Table 1. CSF fungal culture confirmed *Cryptococcus neoformans*. She underwent an excision of the right foot cystic lesion. Unfortunately, no tissue culture was obtained as clinical suspicion of infection was low based on clinical and radiological findings. However, histopathology shows fibro adipose tissue with abscess formation and granulomatous, GMS fungal stain positive, and it shows yeast form fungi with pseudo-hyphae favor Cryptococcus infection (Figure 4).

Initially, oral flucytosine 25 mg/kg q6hr and amphotericin b lipid complex 5 mg/kg intravenous (IV) daily was initiated, but she started having severe allergic reaction from amphotericin B lipid complex, so it was switched to fluconazole 600 mg daily with continuation of oral flucytosine for six weeks, followed by fluconazole 400 mg daily as consolidation and maintenance therapy for one year. Mycophenolate dose was reduced due to disseminated invasive cryptococcal fungal infection and tacrolimus dose was adjusted due to fluconazole drug interactions. She had a good clinical response to the antifungal treatment, and the serial cryptococcus antigen decreased appropriately at 15 months (Table 1). Serum tacrolimus level, liver, and renal function were monitored closely during the treatment. She has successfully finished the 15 months of antifungal therapy and continues to follow up in the clinic for close monitoring of infection recurrence.

## Discussion

Invasive fungal infections are life-threatening infections, accounting for 15-42% of organ transplant recipients [6]. Cryptococcosis is the third leading invasive fungal infection in organ transplant recipients, after candidiasis and aspergillosis, and accounts for 8% of cases. Cutaneous cryptococcus infection is more common in solid organ transplant (SOT) patients compared to hematopoietic stem cell transplant patients, for reasons unknown. Generally, cryptococcus infection is a late-stage post-transplant infection, occurring with the median time to onset ranging from 16 to 21 months post‐transplantation [6-8]. In our case, it occurred seven and a half years after the renal transplantation.

Cutaneous cryptococcus infection presents with a wide variety of skin manifestations ranging from skin papule, umbilical type lesion mimicking Molluscum contagiosum, maculopapular, violaceous nodular lesion, cellulitis, or even cold abscess. Frequently there is a discharging sinus that connects to the underlying deep abscess, or the underlying bone mimics bacterial infection is also seen [4,5]. In a large prospective cohort study of 146 patients with cryptococcosis, the common skin presentations were skin papules, nodular masses, ulcers/abscesses, and cellulitis. Two-thirds of the skin lesions were found in the lower extremities, and 69.2% of cases had disseminated disease. The CNS was involved in 90% of cases with disseminated disease [9]. Ariyoshi et al. recently reported a case of cutaneous cryptococcus with a large giant subcutaneous nodule 14 x 8 cm in size at the thigh with pulmonary cavitation in an immunocompromised host, but MRI shows diffuse edema changes instead of localized cystic mass [10]. Cho et al. reported a case of cutaneous cryptococcus abscess involving the thigh with a mass of 15 cm in size in a kidney transplant recipient patient [11]. Chakradeo et al. reported a case of pruritic rash superimposed on cellulitis of the legs with CNS involvement in a kidney transplant recipient patient [4]. Compared to these cases, our patient had a large multiloculated septated cystic mass 6 x 6 cm in size that was easily visible and palpable, which seems to be a rare presentation based on our literature search. 

Diagnosis of localized cutaneous cryptococcus infection and/or disseminated cryptococcus infection requires serum cryptococcal antigen, blood cultures, CT scan of the lungs, CT scan or MRI of the brain, spinal fluid studies, skin lesion biopsy, and sometimes bronchoscopy or intervention-guided percutaneous lung lesion biopsy for culture and pathology [3,12]. It is very important to submit skin lesion biopsy samples for appropriate cultures as well as for histopathology. Our patient has a cutaneous large cystic lesion, pulmonary cavitary lesion, and cryptococcal meningitis. 

Treatment of cryptococcus infection depends upon localized vs disseminated infection and the immune status of the host. Generally, severe cases with CNS involvement or disseminated infection in organ transplant patients are treated with amphotericin IV along with oral flucytosine for at least two weeks followed by oral fluconazole therapy for up to one year. Mild to moderate infection without CNS involvement and disseminated infection are treated with oral fluconazole for 6-12 months duration. Fluconazole is the treatment of choice, but other azoles like itraconazole, voriconazole, posaconazole, and isavuconazole can be used as an alternative or as salvage therapy [13]. It is very important to monitor drug-drug interaction, especially calcineurin inhibitors (tacrolimus, cyclosporin, or sirolimus) with azoles in transplant patients, along with a reduction in immunosuppressive therapy and expert infectious diseases consultation can be sought for these patients. Treatment details can be found elsewhere [13-15].

Our patient was initially treated with amphotericin B lipid complex 5 mg/kg IV with oral flucytosine 25 mg/kg q6hrs, but unfortunately, she developed a severe allergic reaction, so it was switched to oral high-dose fluconazole 600 mg daily with continued flucytosine for six weeks as induction therapy, followed by oral fluconazole 400 mg as consolidation, then maintenance treatment for one year. The right foot cystic mass required excision, and no recurrence of foot mass was noted. At subsequent follow-up visits, she was clinically improved, and serial cryptococcus antigen decreased appropriately at 15 months. She has successfully finished 15 months of the antifungal therapy and continues to follow up in the clinic for close monitoring of infection recurrence.

## Conclusions

Cryptococcus infection causes localized skin lesions to systemic disseminated infections. Cutaneous cryptococcus infection presents with a wide variety of skin manifestations, but a large cystic painless lesion is a rare manifestation of cutaneous cryptococcus, as in our case. Immunocompromised hosts, including organ transplant recipient patients, always think of indolent fungal infection as a rare cause of cystic cutaneous lesion that warrants further testing, including tissue culture, histopathology, and imaging to look for systemic dissemination of cryptococcus. Prompt diagnosis and appropriate treatment of disseminated cryptococcus infection are essential to improve clinical outcomes.
